# Supplementary material for: Resistance exercise training for anxiety and worry symptoms among young adults: a randomized controlled trial
Source: Sci Rep. 2020 Oct 16;10:17548. doi: 10.1038/s41598-020-74608-6 (PMC7567848; doi:10.1038/s41598-020-74608-6)
Supplement: Supplementary file 2 — Supplementary Information 2. [file 41598_2020_74608_MOESM2_ESM.docx]

**Supplement 1.** Sensitivity Analyses of Changes in Anxiety and Worry Symptoms

| Outcome | Primary Analyses  Hedges’ *d* (95%CI) | Intention to Treat Hedges’ *d* (95%CI) | Complete Cases Only Hedges’ *d* (95%CI) |
| --- | --- | --- | --- |
| Anxiety Symptoms(STAI-Y2) | 0.85 (0.06 to 1.63) | 0.82 (0.21 to 1.44) | 0.93 (0.08 to 1.78) |
| Worry Symptoms (PSWQ) | -0.22(-0.96 to 0.53) | -0.24 (-0.84 to 0.35) | 0.10 (-0.90 to 0.71) |
| Worry-Engagement (PSWQ-WE) | -0.20(-0.94 to 0.54) | -0.12 (-0.72 to 0.47) | -0.16 (-0.96 to 0.65) |
| Absence of Worry (PSWQ-AW) | -0.18(-0.92 to 0.57) | -0.23 (-0.82 to 0.37) | 0.08 (-0.72 to 0.88) |
| STAI-Y2=Trait Anxiety Inventory; PSWQ=Penn State Worry Questionnaire; PSWQ-WE=Penn State Worry Questionnaire-Worry Engagement; PSWQ-AW=Penn State Worry Questionnaire-Absence of Worry. | | | |
